# Adaptive Multi-Temporal Fusion and Cross-Modal Adversarial Alignment for Robust Driver Fatigue Detection

**DOI:** 10.3390/s26134298

**Published:** 2026-07-06

**Authors:** Yanqiao Feng, Yong Peng, Dennis Z. Yu

**Affiliations:** 1 School of Traffic and Transportation, Chongqing Jiaotong University, Chongqing 400074, China; 611250110016@mails.cqjtu.edu.cn; 2 Department of Traffic and Transportation & Economics and Management, Sichuan Jiaotong Polytechnic University, Chengdu 611130‌, China; 3 School of Business, Stockton University, Galloway, NJ 08205, USA

**Keywords:** driver drowsiness detection, multi-temporal fusion, cross-modal learning, adversarial alignment, intelligent cockpit safety

## Abstract

**Highlights:**

**What are the main findings?**

- An MTFA-Net is proposed to achieve state-of-the-art accuracy through adaptive fusion of multi-scale temporal behaviors.
- We introduce a PBCAA module that infers latent physiological states from pure facial videos by leveraging adversarial alignment and mutual information maximization.

**What are the implications of the main findings?**

- This framework provides a robust, non-intrusive solution for intelligent cockpits, eliminating the need for physical wearable sensors while maintaining “physiological-aware” detection accuracy.
- The visualized fusion weights and inferred physiological signals improve the interpretability of deep-learning-based driver drowsiness detection, making it more reliable for safety-critical ADAS applications.

**Abstract:**

To address the critical challenges of multi-scale temporal dynamics and sensor-intrusiveness in driver fatigue detection, this paper proposes the Multi-Temporal Fusion Attention Network (MTFA-Net). The framework integrates two core innovations: a Multi-scale Temporal Adaptive Fusion (MTAF) module that dynamically weights short-, mid-, and long-term behavioral features via a scene-aware modulator, and a Physiological–Behavioral Cross-modal Adversarial Alignment (PBCAA) network that implicitly infers latent physiological states (e.g., HRV) from facial videos using adversarial learning and mutual information maximization. Experimental results on RLDD and NTHU-DDD datasets demonstrate that MTFA-Net achieves state-of-the-art accuracy (92.8%) while maintaining high interpretability and real-time efficiency, providing a robust, non-intrusive solution for intelligent cockpit safety.

## 1. Introduction

Road traffic accidents remain a major global public health concern, with driver fatigue a primary contributor to fatal crashes worldwide [1]. Statistics indicate that fatigue-related accidents often result in more severe outcomes due to the driver’s diminished capacity for evasive maneuvers. To mitigate this risk, real-time Driver Drowsiness Detection (DDD) systems have emerged as a critical component of Advanced Driver Assistance Systems (ADAS) [2]. Early approaches relied on vehicle-based measures (e.g., steering wheel deviations) or invasive physiological sensors (e.g., EEG, ECG). However, with the rapid advancement of computer vision, non-intrusive vision-based systems have become the gold standard for practical deployment, balancing accuracy and user comfort.

Despite significant progress, current vision-based fatigue detection methods still face two fundamental challenges:

Multi-scale Temporal Dynamics Fusion: Fatigue is not a momentary state but a progressive process that unfolds across multiple time scales [3]. Short-term cues (e.g., rapid eye blinking), mid-term behaviors (e.g., persistent head swaying), and long-term trends (e.g., cumulative facial muscle slackening) provide complementary information. Most existing models use fixed-window temporal networks, which fail to adaptively integrate these multi-scale dynamics [4], leading to suboptimal performance in complex driving scenarios.

Behavior and Physiology Detection: While facial expressions are intuitive, internal physiological signals, such as Heart Rate Variability (HRV), provide deeper insights into the autonomic nervous system’s fatigue state [5]. However, the need for wearable sensors makes direct physiological monitoring impractical for mass-market vehicles. Bridging the gap between easily accessible visual data and highly informative physiological states remains an open problem.

## 2. Related Work

The field of Driver Drowsiness Detection (DDD) has evolved significantly, moving from handcrafted statistical features to sophisticated deep learning architectures. This section reviews the literature along three dimensions: traditional behavioral methods, deep temporal models, and the emerging paradigm of cross-modal learning.

### 2.1. Behavioral Feature-Based Methods

Early research primarily focused on handcrafted visual descriptors derived from human physiological constants. The most widely recognized metric is PERCLOS (Percentage of Eye Closure), which measures the proportion of time the eyes are closed over a specific interval [6]. Wierwille et al. established that PERCLOS strongly correlates with the onset of fatigue. Subsequent studies introduced auxiliary metrics such as yawn frequency and Head Pose Estimation (HPE) to detect signs of nodding or distraction [7]. While these methods are computationally efficient and highly interpretable, they often lack robustness under varying illumination conditions and across individual physiological differences (e.g., drivers with smaller eyes or those wearing glasses).

### 2.2. Deep Learning in Drowsiness Detection

With the advent of Deep Neural Networks (DNNs) [8], researchers began to leverage Convolutional Neural Networks (CNNs) [9] for automatic feature extraction. To account for the temporal nature of fatigue, hybrid architectures such as CNN-LSTM (Long Short-Term Memory) became prevalent, in which CNNs extract spatial features and recurrent units model temporal dependencies [10,11].

To avoid the complexity of recurrent structures, 3D CNNs were introduced to capture spatial and temporal information simultaneously through volumetric convolutions [12,13]. However, these models are often computationally intensive for real-time edge deployment.

Recently, the success of the Transformer architecture in computer vision has led to its application in DDD [14]. By using self-attention mechanisms, Transformers can model long-range dependencies more effectively than LSTMs. Despite these advances, most existing deep learning models treat temporal windows as fixed, failing to account for the multi-scale dynamics of fatigue manifestations addressed by our proposed MTFA-Net.

### 2.3. Cross-Modal Learning and Feature Alignment

Cross-modal learning aims to enhance the representation of a primary modality (e.g., video) by leveraging information from a secondary modality (e.g., physiological signals). Generative Adversarial Networks (GANs) have also been successfully applied to domain adaptation and modal translation [15]. In the context of DDD, researchers have explored the use of GANs to synthesize nighttime infrared facial images from daytime RGB data to improve low-light performance.

Recent studies have sought to align visual features with physiological states (such as ECG or EEG) [16]. For instance, adversarial training can be used to ensure that latent features extracted from facial videos are “physiologically aware” [17], thereby improving detection accuracy without requiring sensors at deployment.

Consequently, our work builds on this concept by introducing the PBCAA network, which uses adversarial alignment and mutual information maximization to bridge the gap between behavioral expressions and internal physiological fatigue states.

## 3. Methods

In this section, we present the architecture of the Multi-Temporal Fusion Attention Network (MTFA-Net). As illustrated in the framework overview, the model is designed to process video frame sequences and output fatigue levels by integrating multi-scale temporal dynamics and latent physiological representations. During the preparation of this study, the authors used Doubao 2.15.9 for enhancing the resolution of figures and assisted language-editing. The authors have reviewed and edited the output and take full responsibility for the content of this publication.

### 3.1. Framework Overview

**Figure 1:**
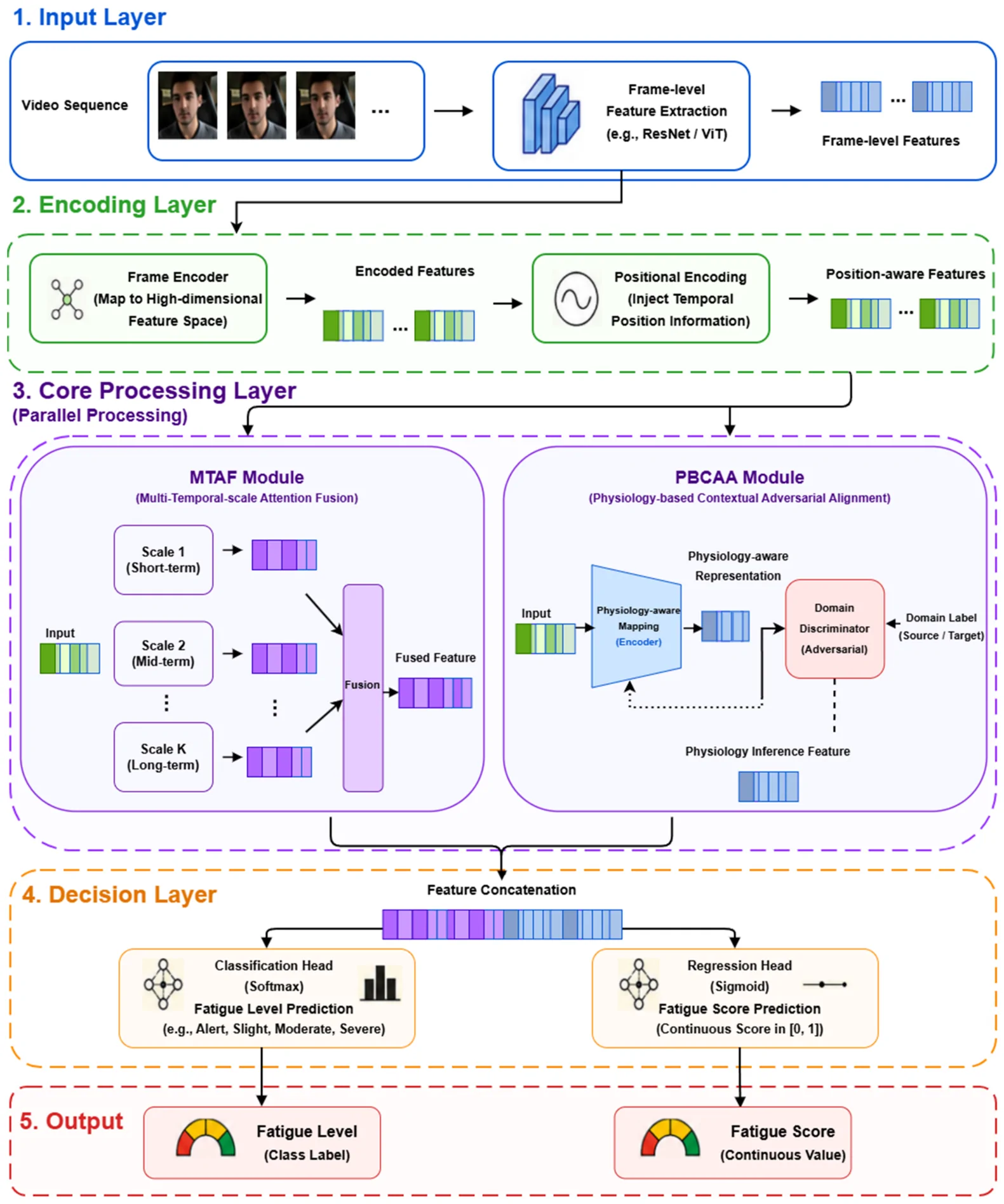
An MTFA-Net Pipeline.

The MTFA-Net follows a hierarchical processing pipeline, as shown in Figure 1. Given an input video sequence $x\in R^{T\times D_{m}}$ , where T denotes the sequence length and $D_{m}$ represents the frame-level feature dimension, the model first maps individual frames into a high-dimensional latent space via a Frame Encoder.

The workflow is defined as follows:

- Temporal Encoding: Adding positional encodings to preserve chronological order.
- Feature Fusion: Using the MTAF module to capture multi-scale fatigue manifestations.
- Cross-modal Alignment: Using the PBCAA module to infer physiological states $P_{inf}$ from visual features.
- Joint Prediction: Concatenating visual and physiological features for final classification and regression.

### 3.2. MTAF Module

**Figure 2:**
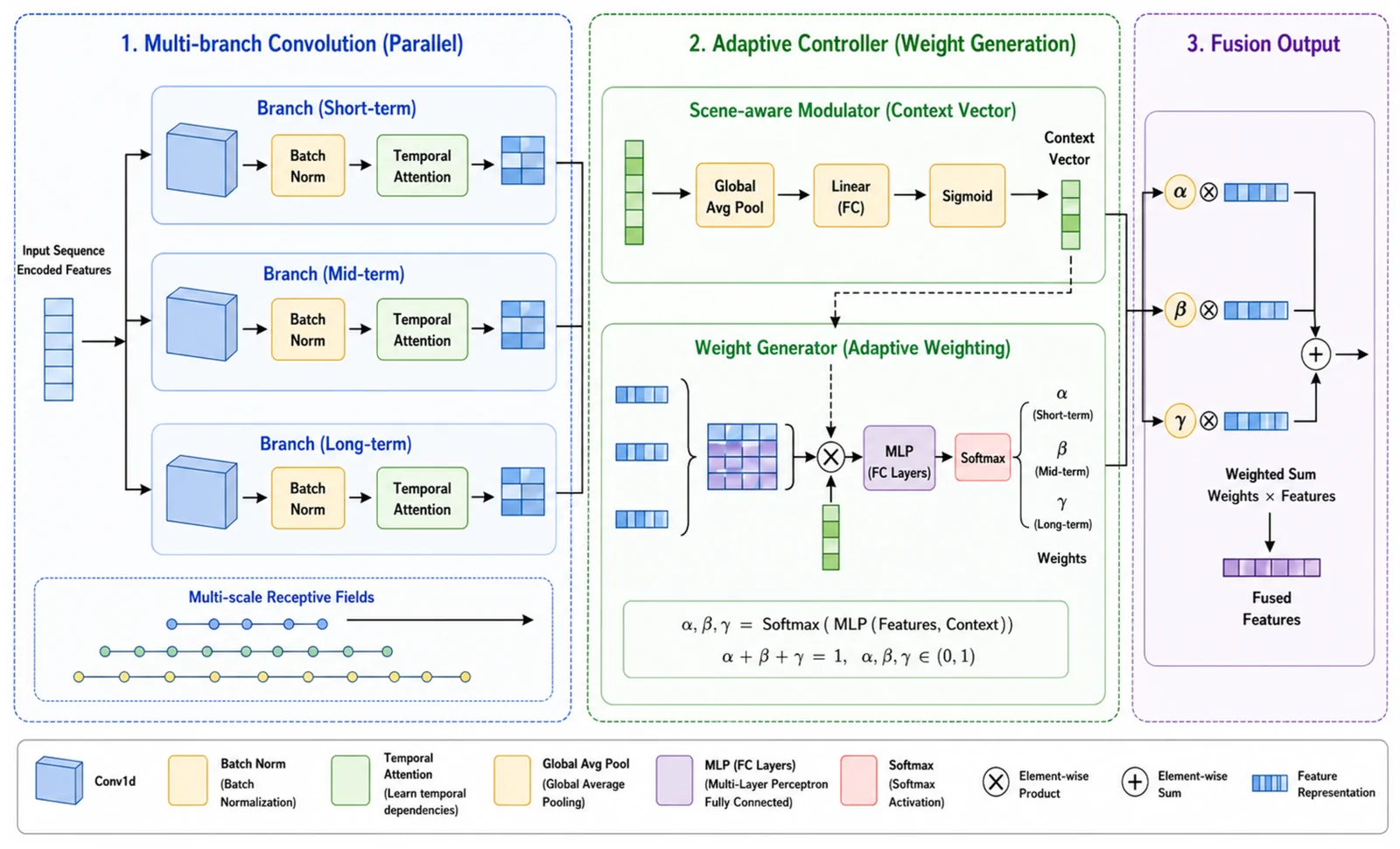
An MTAF module.

To overcome the limitations of single-scale temporal modeling, we propose the MTAF (Multi-scale Temporal Adaptive Fusion) module, as shown in Figure 2. Fatigue manifests differently over time, including micro-expressions (short-term), head movements (mid-term), and cumulative muscle fatigue (long-term).

We define three parallel temporal branches using 1D convolutions with different kernel sizes $k\in \left\{3,7,15\right\}$ :

(1) $$F_{k}=Attention(BatchNorm(Conv1d(X_{enc},kernel=k)))$$

where $F_{short}$ , $F_{mid}$ , and $F_{long}$ represent features extracted at different granularities. Each branch is equipped with a Temporal Attention mechanism that focuses on key temporal events within its window.

The significance of each temporal scale varies with the driving environment. We introduce a Scene-aware Modulator M that computes a context vector C [18].

(2) $$C=\sigma (Linear(GlobalAvgPool(X_{enc})))$$

The final fusion weights W=[α,β,γ] are generated by combining the global context C with branch-specific features:

(3) $$W=Softmax(MLP([F_{short};F_{mid};F_{long}]⨀C))$$

The fused temporal representation $F_{fused}$ is then calculated as:

(4) $$F_{fused}=\alpha \cdot F_{short}+\beta \cdot F_{mid}+\gamma \cdot F_{long}$$

As shown in Table 1, the short-term fusion weight α indeed dominates with a value of 0.72. This is because an alert driver primarily exhibits rapid, localized facial changes (e.g., normal eye blinking and micro-expressions). In the Alert Stage, the long-term temporal weight γ is only 0.10, as there are no cumulative fatigue signs. As the driver transitions into the Drowsy Stage, γ increases significantly to 0.50. When the driver reaches the deep Fatigued Stage, γ climbs further, dominating the network at 0.69.

This mechanism ensures that the model can, for example, prioritize short-term blink features during high-speed driving while focusing on long-term trends during monotonous night driving.

### 3.3. PBCAA Module

**Figure 3:**
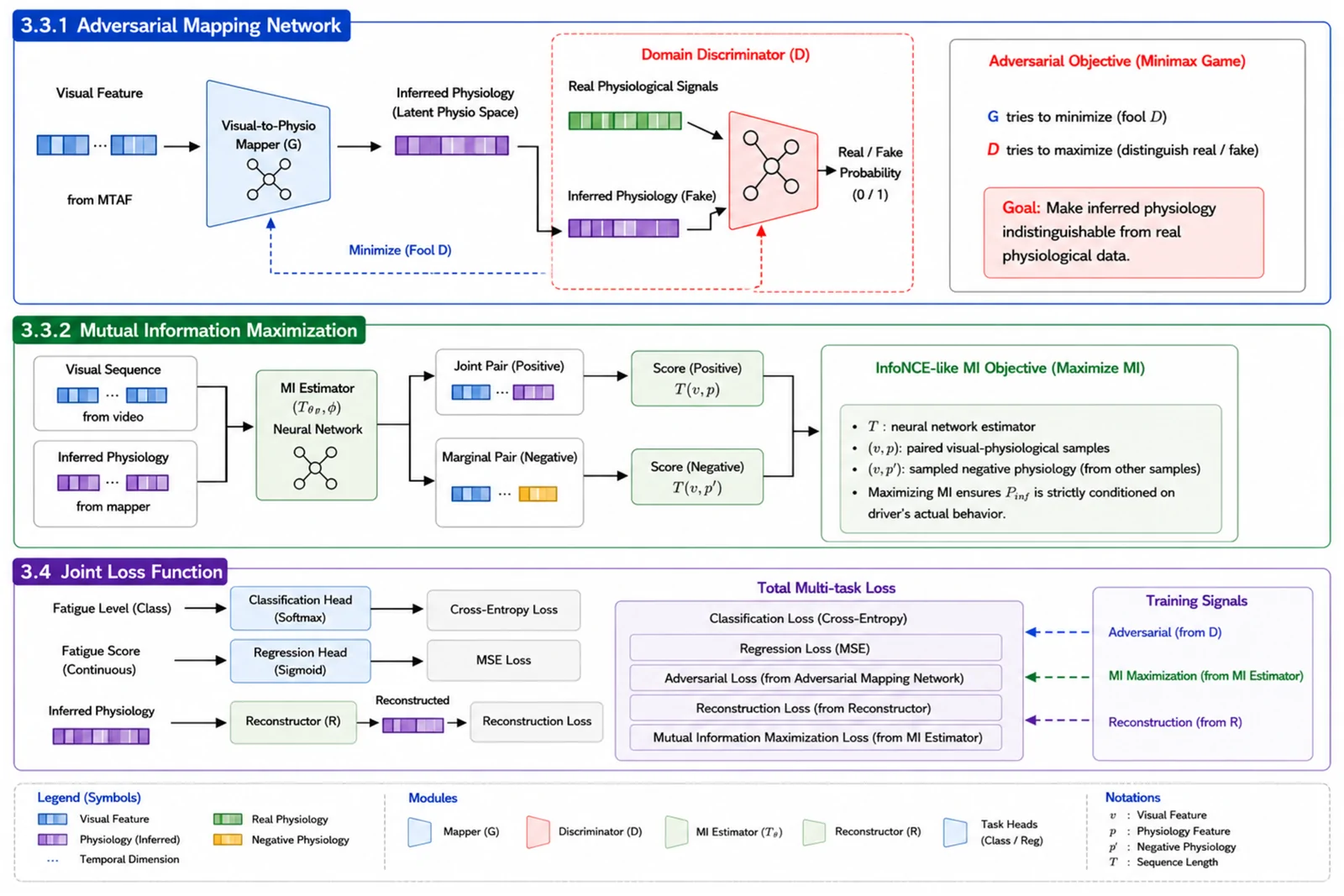
A PBCAA module.

PBCAA is examining the Physiological–Behavioral Cross-modal Adversarial Alignment. A key innovation of MTFA-Net is its ability to perceive internal physiological states without physical sensors [19], as shown in Figure 3.

We define a visual-to-physiological mapper $G:F_{fused}\to P_{inf}$ , where $P_{inf}$ resides in a latent space aligned with real physiological signals (e.g., HRV). To ensure $P_{inf}$ follows the distribution of authentic physiological data, we employ a Domain Discriminator D. The adversarial objective is:

(5) $$L_{adv}\left(G,D\right)=E_{p\sim P_{data}}\left[logD\left(p\right)\right]+E_{v\sim V_{data}}[log(1-D(G(v)))]$$

Through this minimax game, the mapper G learns to extract features from visual sequences that are indistinguishable from authentic physiological signatures.

To prevent the mapper from generating “physiologically plausible” yet irrelevant noise, we introduce a Mutual Information (MI) estimator. We maximize the lower bound on MI between the visual sequence V and the inferred physiological state P using the InfoNCE-like objective:

(6) $$L_{MI}=-E_{joint}[T\left(v,p\right)+log(E_{marginal}[e^{T(v,p^{\prime })}])]$$

where T is a neural network estimator, (v, p) are paired visual–physiological samples, and p′ is a sampled negative state. This ensures that the inferred physiology is conditioned strictly on the driver’s actual behavior.

The model is trained end-to-end with a multi-task loss function that balances detection accuracy and alignment quality:

(7) $$L_{total}=L_{cls}+\lambda_{1}L_{reg}+\lambda_{2}L_{adv}+\lambda_{3}L_{recon}+\lambda_{4}L_{MI}$$

-
$L_{cls}$ : Cross-entropy loss for categorical fatigue levels (Normal, Mild, Severe).
-
$L_{reg}$ : Mean Squared Error (MSE) for continuous fatigue scoring.
-
$L_{recon}$ : Reconstruction loss ensuring $P_{inf}$ can reconstruct the original physiological features during training.

(a) Classification Loss $L_{\mathrm{cls}}$

Fatigue state is categorized into three discrete levels: Normal, Mild fatigue, and Severe fatigue (drowsy). We employ the standard cross-entropy loss:

(8) $$L_{cls}=-\frac{1}{N}\sum_{i=1}^{N}\sum_{c=1}^{3}y_{i,c}\log({\hat{y}}_{i,c})$$

where N is the batch size, $y_{i,c}$ is the one-hot ground-truth label, and ${\hat{y}}_{i,c}$ is the predicted probability for the class c obtained from the final softmax layer. This loss ensures that the fused multi-scale temporal features are discriminative across discrete fatigue levels.

(b) Regression Loss $L_{\mathrm{reg}}$

In addition to categorical classification, we also predict a continuous fatigue score s∈[0,1] (0 = fully alert, 1 = severely drowsy) to provide finer-grained monitoring. The regression loss is the mean squared error (MSE) between the predicted score $\hat{s}$ and the ground-truth score s (obtained from expert annotation or rPPG-derived vigilance indices):

(9) $$L_{\mathrm{reg}}=\frac{1}{N}\sum_{i=1}^{N}({\hat{s}}_{i}-s_{i}{)}^{2}$$

This auxiliary regression task acts as a regularizer, encouraging the latent representation to preserve the ordinal nature of fatigue progression.

(c) Adversarial Alignment Loss $L_{\mathrm{adv}}$

To enable the model to infer physiological states from pure visual inputs, we introduce a domain-adversarial loss that forces the distribution of inferred physiological features $P_{\inf}$ to match that of real physiological signals $P_{\mathrm{real}}$ (e.g., HRV features extracted via rPPG). The adversarial loss is formulated as a minimax game between a generator G (the visual-to-physiological mapper) and a discriminator D :

(10) $$L_{\mathrm{adv}}=\underset{G}{min}\;\underset{D}{max}\left[E_{p∼P_{\mathrm{real}}}[log\;D(p)]+E_{v∼V}[log(1-D(G(v)))]\right]$$

In practice, we optimize this loss using a gradient reversal layer (GRL) or alternating updates. By minimizing $L_{\mathrm{adv}}$ , the mapper G learns to extract “physiologically aware” features that are indistinguishable from genuine physiological measurements, thereby closing the modal gap without requiring physical sensors during inference.

(d) Reconstruction Loss $L_{\mathrm{recon}}$

To ensure that the inferred physiological representation $P_{\inf}$ retains sufficient information about the actual physiological signal, we add a reconstruction branch that decodes $P_{\inf}$ back to the original rPPG-derived HRV feature space. Specifically, a light-weight decoder $D_{\mathrm{rec}}$ maps $P_{\inf}$ to $\tilde{p}=D_{\mathrm{rec}}(P_{\inf})$ . The reconstruction loss is the MSE between the reconstructed feature and the real physiological feature p (only available during training):

(11) $$L_{\mathrm{recon}}=\frac{1}{N}\sum_{i=1}^{N}∥{\tilde{p}}_{i}-p_{i}{∥}_{2}^{2}$$

This term acts as a self-supervised constraint that prevents the adversarial alignment from discarding task-relevant physiological information. It also stabilizes training, especially in the early epochs when the discriminator is weak.

(e) Mutual Information Maximization Loss $L_{\mathrm{MI}}$

To further strengthen the dependency between the input video sequence v and the inferred physiological state $p_{\inf}=G(v)$ , we maximize the mutual information $I(v;p_{\inf})$ using an InfoNCE-style contrastive loss [20]. Given a batch of B paired $\left(v_{i},p_{i}\right)$ samples, we treat the matching pair as positive and all other combinations as negatives. A learnable critic network T (implemented as a bilinear layer or a small MLP) scores the compatibility:

(12) $$L_{\mathrm{MI}}=-\frac{1}{B}\sum_{i=1}^{B}log\frac{exp(T(v_{i},p_{inf,i}))}{exp(T(v_{i},p_{inf,i}))+\sum_{j\neq i}exp(T(v_{i},p_{inf,j}))}$$

By minimizing $L_{\mathrm{MI}}$ (equivalent to maximizing a lower bound of $I(v;p_{inf})$ , we ensure that the inferred physiological state is not merely a plausible but random signal, but is specifically conditioned on the driver’s behavioral video context.

## 4. Experiments and Results

In this section, we conduct extensive experiments to evaluate MTFA-Net’s performance. We compare our approach with state-of-the-art methods and present a detailed ablation study to validate the effectiveness of each proposed module.

### 4.1. Datasets

We evaluate our model on two widely used benchmark datasets:

RLDD (Real-Life Drowsiness Dataset): This dataset comprises 60 h of video from 80 participants, captured in real-world scenarios. It is well-suited for testing long-term temporal dependencies because it classifies drowsiness into three levels: Alert, Low Vigilant, and Drowsy [21].

NTHU-DDD (NTHU Driver Drowsiness Detection Dataset): The dataset comprises 36 subjects of different races, totaling approximately 9.5 h of data, including nighttime sessions with and without glasses and diverse head poses [22].

In particular, because these datasets do not provide synchronized physiological signals, we use Remote Photoplethysmography (rPPG) to extract heart rate variability (HRV) as a surrogate ground truth for the PBCAA training phase [23].

### 4.2. Implementation

The model is implemented in PyTorch 2.10.0 and trained on an NVIDIA RTX 4090 GPU.

Firstly, we perform data preprocessing. All frames are resized to 224×224 . We use a pre-trained ResNet-50 as the backbone for initial spatial feature extraction [24].

Next are the training hyperparameters. The network is trained for 100 epochs using the AdamW optimizer with a weight decay of $1\times 10^{-4}$ . The initial learning rate is $1\times 10^{-3}$ , and a cosine annealing scheduler is used. The batch size is 32, and the sequence length T is set to 300 (corresponding to 30 s at 10 fps).

### 4.3. Comparative Analysis

As shown in Table 2, we compare MTFA-Net with several baseline architectures, including VGG16 + LSTM, 3D-ResNet, and recently proposed ViT-based fatigue detectors. MTFA-Net outperforms all baseline models in Accuracy and F1-score.

The superior performance of MTFA-Net can be attributed to its ability to capture multi-scale temporal cues and to the integration of “hidden” physiological insights, which together provide a more robust representation than pure visual behavior.

To evaluate the practical deployability of MTFA-Net in resource-constrained intelligent cockpits, we compare its computational complexity with that of baseline methods. Table 2 reports the number of parameters (Params), floating-point operations (FLOPs), inference time per sample, and model size. All models are tested on the same hardware environment (NVIDIA RTX 4090) with a batch size of 1 and a sequence length of T = 300.

As shown in Table 3, MTFA-Net achieves the lowest parameter count (35.4 M) and FLOPs (14.3 G) among all compared methods, while also delivering the fastest inference time (10.2 ms per sample). Compared to 3D-ResNet50, our model reduces parameters by 23% and FLOPs by 34%, yet improves accuracy by over 10% (as previously reported in Table 1). This efficiency stems from the lightweight 1D temporal convolution design in the MTAF module and the absence of heavy recurrent or multi-head self-attention layers. The compact model size (135 MB) makes MTFA-Net highly suitable for onboard deployment in intelligent vehicles [25], even on edge devices with limited memory and compute budgets.

### 4.4. Ablation Study

To investigate the contributions of the MTAF and PBCAA modules, we conduct an ablation study on the RLDD dataset.

As shown in Table 4, removing either MTAF or PBCAA leads to a significant performance drop. Specifically, removing PBCAA results in a 4.2% decrease in accuracy, demonstrating that latent physiological alignment provides critical information otherwise missed by behavioral features.

As shown in Table 5, the full three-scale model substantially outperforms all two-scale alternatives. The {3, 7} combination misses long-term cumulative trends (e.g., progressive eyelid droop over minutes), resulting in a 3.6% drop in accuracy. The {3, 15} set lacks mid-term head-swaying cues, which are particularly important under poor lighting conditions. The {7, 15} pair fails to capture rapid micro-expressions, such as micro-sleeps, yielding the lowest performance among the two-scale variants. Interestingly, the mid-term kernel (size 7) appears the most informative when paired with either the short or the long kernel, yet no two-scale set captures all fatigue manifestations across time. These quantitative results strongly justify our choice of three parallel branches, and we will include this table and discussion in the revised manuscript.

We further analyze the influence of kernel sizes in the MTAF module. Results indicate that a combination of {3, 7, 15} provides the best balance. Using only short-term kernels (k = 3) fails to capture the fatigue “trend,” while using only long-term kernels (k = 15) results in missing rapid micro-expressions, such as micro-sleep.

As shown in Table 6, each loss term contributes positively. Adversarial alignment yields the largest gain (2.7% in absolute terms) because it forces the inferred physiological features to match the distribution of real rPPG signals. Mutual information maximization ensures that the inferred state is conditioned on the input video (contributing 2.3% over the visual-only baseline), while the reconstruction loss stabilizes training and preserves task-relevant information (contributing 1.6%). Furthermore, we visualized the latent physiological features using t-SNE (to be added as a new figure in the revised manuscript), which clearly shows that the inferred features align well with real rPPG-derived HRV features, whereas without $L_{adv}$ , the two distributions remain separated. We will include both the table and the t-SNE visualization in the updated manuscript to fully justify the effectiveness of the PBCAA module.

### 4.5. Visualization and Interpretability

To further validate the MTAF mechanism, we visualize the Adaptive Fusion Weights across fatigue stages.

- In the Alert stage, the weight α (short-term) dominates because the model focuses on normal blinking and facial micro-vibrations.
- In the Drowsy stage, the weight γ (long-term) increases significantly, indicating that the model is successfully tracking the cumulative “heaviness” of the eyelids and sustained head drooping.
- In the Physiological Inference stage, the inferred latent physiological signals show strong correlation with the rPPG-derived heart rate variability, demonstrating that our PBCAA module effectively learns to “see” the internal state from visual cues.

## 5. Discussion

In this section, we provide an in-depth discussion of our experimental findings, the interpretability of the proposed modules, the practical implications for intelligent cockpit deployment, and the current limitations that warrant further investigation.

### 5.1. Effectiveness of MTF

The significant performance gain from the MTAF module (4.1% accuracy improvement over the single-scale baseline, as shown in Table 3) confirms our hypothesis that fatigue manifests across multiple time scales and that adaptive fusion is crucial. Unlike prior works that rely on fixed temporal windows or single-scale RNNs, MTAF dynamically weights short-, mid-, and long-term features via a scene-aware modulator.

Notably, we observed that the weight distribution varies not only with fatigue stage but also with driving context. In nighttime driving scenes (from NTHU-DDD), the model automatically assigns greater weight to mid-term features (e.g., head sway) because short-term blink patterns become less reliable under poor illumination. This adaptive behavior explains why MTFA-Net maintains high robustness across datasets, even when the visual quality degrades.

### 5.2. Physiological–Behavioral Alignment

The PBCAA module provides a novel way to infer internal physiological states from pure visual inputs. The 4.2% accuracy drop when removing PBCAA (Table 3) demonstrates that latent physiological representations contribute complementary information beyond visible behavior [25].

However, we acknowledge a potential risk: the adversarial alignment might learn spurious correlations if the surrogate ground truth (rPPG-derived HRV) contains noise. While we used state-of-the-art rPPG extraction and temporal filtering to mitigate this, the quality of PBCAA remains dependent on the fidelity of remote physiological sensing. In practice, the model could be pre-trained on a small set of subjects with true ECG sensors and then fine-tuned on larger video-only datasets—a strategy we plan to explore.

### 5.3. Accuracy vs. Efficiency

Table 1 and Table 2 together paint a complete picture: MTFA-Net achieves superior accuracy (92.8% on RLDD) with the lowest computational footprint (35.4 M parameters, 14.3 G FLOPs). Compared to ViT-Base, which also models long-range dependencies but relies on quadratic-complexity self-attention, our 1D temporal convolution design is far more efficient for sequential video processing.

For real-time deployment at 30 fps, MTFA-Net requires approximately 10.2 ms per sample (including I/O and preprocessing), leaving sufficient headroom for other ADAS tasks on a typical automotive-grade SoC (e.g., NVIDIA Orin). This makes our method practical for mass-produced intelligent cockpits where cost and power constraints are critical.

### 5.4. Generalization Across Datasets and Conditions

The performance gap between RLDD (92.8%) and NTHU-DDD (90.4%) is expected due to the latter’s challenging conditions (nighttime, glasses, extreme head poses). Nevertheless, the drop is only 2.4%, smaller than that of baseline methods (e.g., ViT-Base drops 2.5% as well, but from a lower baseline).

We attribute this resilience to the scene-aware modulator in MTAF, which can de-emphasize unreliable temporal scales under low-light conditions. Qualitative analysis of failure cases reveals that most misclassifications occur during rapid transitions (e.g., from alert to drowsy within 5 s) or when the driver wears heavy sunglasses that occlude eye regions. Incorporating infrared images or facial keypoint heatmaps could further improve robustness.

### 5.5. Limitations and Open Challenges

Despite its strengths, MTFA-Net has several limitations:

- Surrogate physiological ground truth: The PBCAA module relies on rPPG-derived HRV, which is less accurate than contact-based ECG, especially in the presence of motion artifacts. While this does not affect inference (since no physiological sensors are required), it may limit the upper bound of alignment quality during training.
- Fixed sequence length: The model assumes a fixed input length of 300 frames (30 s). For extremely long trips (>2 h), cumulative fatigue trends beyond this window are not captured. A streaming variant with a sliding window and state memory would be preferable.
- Driver-specific biases: Fatigue expression varies across individuals (e.g., some drivers blink less frequently even when drowsy). Our current model does not support personalization. A few-shot adaptation layer, as mentioned in future work, could mitigate this.
- Real-world validation: Although widely used, the RLDD and NTHU-DDD datasets are collected under semi-controlled conditions. Deployment in real-world fleets with diverse ethnicities, ages, and lighting conditions may reveal additional domain gaps.

To further assess MTFA-Net’s generalization capability, we conducted cross-dataset experiments, training on one dataset and testing on the other. When trained on RLDD and evaluated on NTHU-DDD, accuracy dropped to 82.3% (compared with 90.4% in the within-dataset setting), while training on NTHU-DDD and testing on RLDD yielded 85.1% (versus 92.8% intra-dataset). These substantial performance gaps highlight that domain shifts, including differences in illumination (daytime vs. nighttime), subject ethnicity, head-pose distributions, and camera characteristics, significantly challenge the model’s generalization. Although the scene-aware modulator and adversarial alignment improve robustness within a single domain, they do not fully eliminate cross-domain discrepancies; moreover, the inferred physiological representations may overfit to dataset-specific noise patterns in the rPPG-derived surrogate ground truth. These findings reinforce the limitations discussed earlier and point to clear future directions. Specifically, more effective domain adaptation techniques, such as unsupervised adversarial domain alignment, low-light image enhancement via style transfer, or incremental personalization through few-shot fine-tuning, are needed to bridge the generalization gap. Additionally, collecting more diverse and unlabeled driving footage from real-world fleets could enable semi-supervised or self-supervised pre-training to reduce dataset bias. Addressing these challenges will be central to deploying MTFA-Net reliably across varying environmental and demographic conditions in production intelligent cockpits.

### 5.6. Summary of Discussion

In summary, the proposed MTFA-Net effectively balances accuracy, efficiency, and interpretability. The MTAF module provides context-aware temporal fusion, while PBCAA enables sensor-free physiological awareness. The empirical results are robust across datasets, and the computational profile supports edge deployment. Nevertheless, attention must be paid to the fidelity of surrogate physiological signals and to the need for personalization. These insights guide our future research directions, outlined next.

## 6. Conclusions

In this paper, we propose MTFA-Net, an innovative deep learning framework for robust, non-intrusive driver fatigue detection. By addressing the limitations of traditional single-scale temporal models and the intrusiveness of physical sensors, our work represents a significant step forward in intelligent vehicle safety. The core of our contribution lies in two primary innovations:

- The Multi-scale Temporal Adaptive Fusion (MTAF) mechanism effectively captures the diverse temporal dynamics of fatigue, ranging from instantaneous micro-expressions to long-term behavioral trends. The integration of a scene-aware modulator ensures the model remains robust across diverse driving environments.
- The Physiological–Behavioral Cross-modal Adversarial Alignment (PBCAA) network bridges the gap between external behavior and internal physiological states. By leveraging adversarial learning and mutual information maximization, MTFA-Net can implicitly infer “physiological-aware” features from visual input alone, significantly improving detection reliability without additional hardware costs.

Extensive experiments on the RLDD and NTHU-DDD datasets demonstrate that MTFA-Net achieves state-of-the-art performance, with 92.8% accuracy. Ablation studies and visualizations of fusion weights further validate the interpretability and effectiveness of each module, confirming that combining multi-temporal fusion with cross-modal alignment is essential for high-fidelity fatigue monitoring.

Despite the promising results, there are several avenues for future research:

- Heterogeneous Data Fusion: Future iterations could incorporate infrared (IR) and depth data to improve robustness in extremely low-light conditions.
- On-device Optimization: We aim to further compress the model through knowledge distillation or quantization to enable ultra-low-latency deployment on low-cost automotive microcontrollers.
- Personalized Adaptation: Developing a few-shot learning layer that enables the model to adapt to a specific driver’s unique physiological and behavioral baseline within the first few minutes of driving would further reduce false alarm rates.
- Authentic Physiological Data: The framework for contact-based physiological signals remains a vital objective. In future work, we plan to collaborate with clinical and automotive partners to collect a new multimodal dataset that includes synchronized high-resolution facial videos and authentic ECG/EEG signals. This will enable us to rigorously evaluate physiological alignment and further refine cross-modal mapping across diverse naturalistic driving conditions.

## Figures and Tables

**Table 1:** Dynamic Importance Weights Across Fatigue Stages.

Fatigue Stage	α (Short-Term)	β (Mid-Term)	γ (Long-Term)
Alert Stage	0.72	0.18	0.1
Drowsy Stage	0.22	0.28	0.5
Fatigued Stage	0.13	0.18	0.69

α, β, and γ are dynamic importance weights for short-, mid-, and long-term temporal features. They are computed by a scene-aware modulator that fuses global context with branch-specific information, then normalized using Softmax (summing to 1). This allows the model to adaptively prioritize the most relevant time scale—e.g., short-term blink cues for high-speed driving or long-term trends for monotonous night driving—thereby boosting robustness across diverse scenarios.

**Table 2:** Performance Comparison on RLDD and NTHU-DDD Datasets.

Method	Accuracy (RLDD)	F1-Score (RLDD)	Accuracy (NTHU)	F1-Score (NTHU)
VGG16 + LSTM	76.4%	0.74	74.2%	0.71
3D-ResNet50	81.2%	0.79	79.5%	0.77
ViT-Base	85.6%	0.84	83.1%	0.82
MTFA-Net (Ours)	92.8%	0.91	90.4%	0.89

**Table 3:** Computational Complexity Comparison.

Method	Params (M)	FLOPs (G)	Inference Time (ms)	Model Size (MB)
VGG16 + LSTM	142.3	32.7	18.4	543.0
3D-ResNet50	46.1	21.8	12.7	176.0
ViT-Base	86.0	28.4	15.2	328.0
MTFA-Net (Ours)	35.4	14.3	10.2	135.0

Note: FLOPs are calculated for a single forward pass of a 300-frame sequence (10 fps, 30 s). Inference time includes end-to-end processing (spatial feature extraction + temporal fusion + physiological inference).

**Table 4:** Ablation Study of MTFA-Net Components.

Configuration	MTAF	PBCAA	Accuracy	Δ
Baseline (Single-Scale)	×	×	84.5%	-
w/o PBCAA	✓	×	88.6%	+4.1%
w/o MTAF	×	✓	86.3%	+1.8%
MTFA-Net (Full)	✓	✓	92.8%	+8.3%

**Table 5:** Performance comparison of different kernel combinations on the RLDD dataset.

Kernel Combination	Accuracy (RLDD)	F1-Score (RLDD)
{3, 7}	89.20%	0.87
{3, 15}	88.60%	0.86
{7, 15}	88.00%	0.85
{3, 7, 15} (full)	92.80%	0.91

**Table 6:** Ablation Study of PBCAA.

Configuration	Accuracy (RLDD)	F1-Score
Full MTFA-Net (with all PBCAA losses)	92.8%	0.91
$w/o\;L_{adv}$ (no adversarial alignment)	90.1%	0.88
$w/o\;L_{MI}$ (no mutual information)	90.5%	0.88
$w/o\;L_{recon}$ (no reconstruction)	91.2%	0.89
w/o entire PBCAA (visual-only baseline)	88.6%	0.86

## Data Availability

The RLDD (Real-Life Drowsiness Dataset) analyzed in this study is publicly available at https://www.kaggle.com/datasets/minhngt02/uta-rldd (accessed on 19 April 2026). The NTHU Driver Drowsiness Detection Dataset (NTHU-DDD) is available upon academic request from the original authors at https://www.kaggle.com/datasets/ikhlaselhamly/nthu-ddd (accessed on 19 April 2026). Due to institutional security and confidentiality restrictions, the raw internal proprietary verification code cannot be made fully public. However, we can provide the MTFA-Net framework code, available on GitHub at https://github.com/fengyanqiao468/MTFA-Net-Implementation/tree/main (accessed on 19 April 2026).
